# Interfacial engineering of TiO_2_–WO_3_@MWCNTs nanocomposites for ultrasensitive and highly selective electrochemical nitrite sensing in food samples

**DOI:** 10.1039/d6ra06391h

**Published:** 2026-09-03

**Authors:** Asmaa H. Ragab, Hend S. Mager, Rasha M. El Nashar, Rabeay Y. A. Hassan

**Affiliations:** a Biosensors Research Lab, Zewail City of Science and Technology 6th October City Giza 12578 Egypt ryounes@zewailcity.edu.eg +201129216152; b Applied Organic Chemistry Department, National Research Centre (NRC) Dokki Giza 12622 Egypt hendamer2000@yahoo.com; c Chemistry Department, Faculty of Science, Cairo University Giza 12613 Egypt

## Abstract

Nitrite ions are extensively utilized in the food industry to enhance preservation, flavor, and colorants; however, their excessive intake poses serious health risks, including mutagenic and carcinogenic effects. In this work, a novel electrochemical sensor platform providing high sensitivity and high selectivity was constructed using TiO_2_–WO_3_@MWCNTs-modified carbon paste electrodes (CPEs). The synergistic combination of TiO_2_–WO_3_ bi-metal oxide nanostructures with multi-walled carbon nanotubes significantly enhanced electrochemical reactivity and electrocatalytic activity of the electrode interface. The fabricated nanocomposite was systematically characterized through Fourier-transform infrared spectroscopy (FTIR), transmission electron microscopy (TEM), electrochemical impedance spectroscopy (EIS), and cyclic voltammetry (CV) to identify all acquired features. After assay optimization and mechanistic investigation, the modified electrode exhibited an ultralow detection limit of 0.003 µM and a wide linear detection range of 0.01–210 µM for nitrite detection using chronoamperometry, with excellent selectivity. The sensor's practical applicability was validated through analysis of real food samples, yielding high recovery values and confirming its accuracy and reliability.

## Abstract 2

Schematic workflow of a portable TiO_2_–WO_3_@MWCNTs nanostructured electrochemical platform for sensitive detection and quantification of free nitrite ions (NO_2_^−^) in food samples.

## Graphical abstract

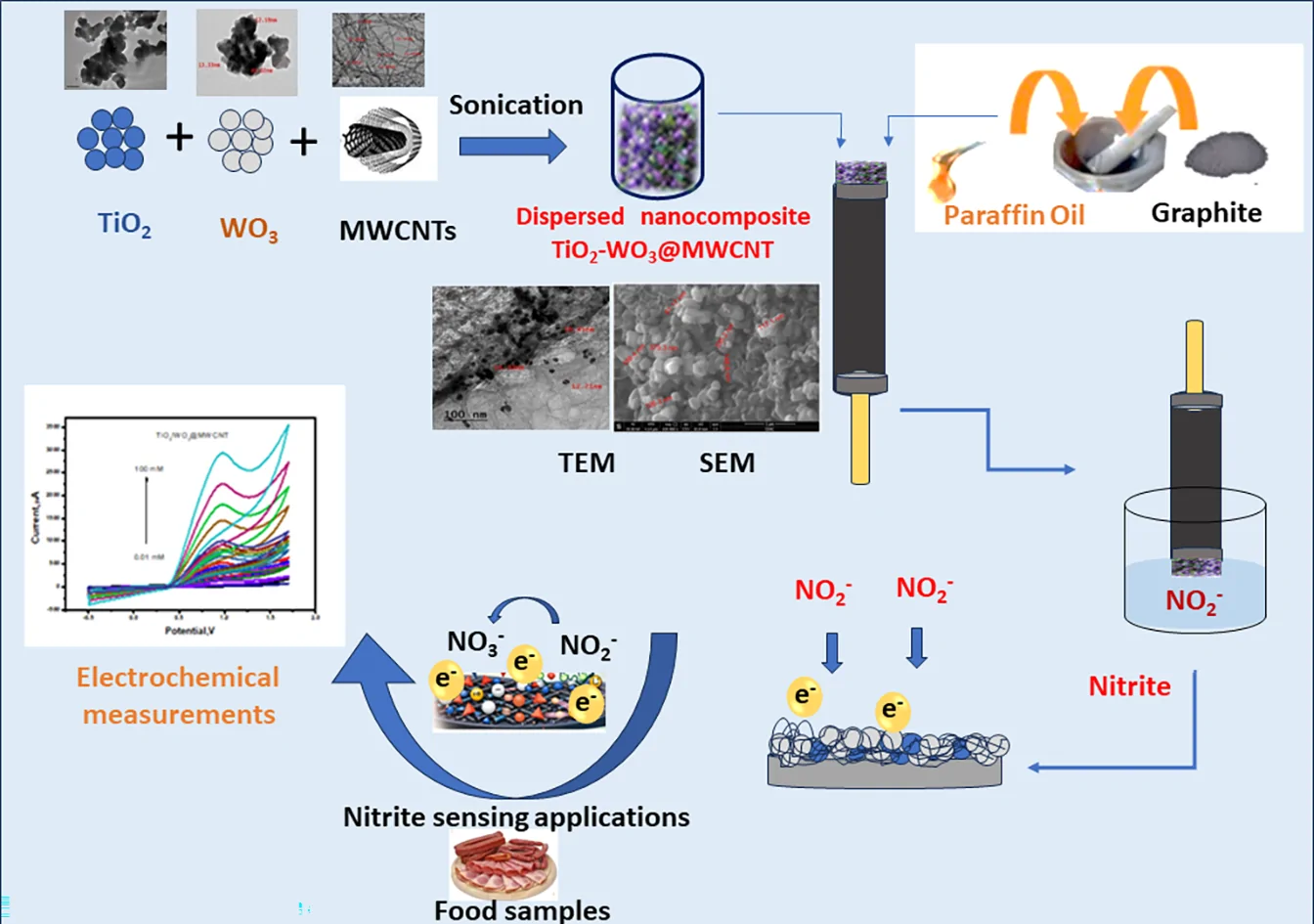

## 1. Introduction

Nitrite-containing compounds are extensively utilized in numerous industrial and commercial sectors, including food preservation, agriculture, textile manufacturing, and pharmaceutical formulations.^1,2^ Despite their broad applicability, excessive exposure to or consumption of nitrite poses significant risks to human health. In this regard, increasing free nitrite levels can induce methemoglobinemia, a pathological condition in which hemoglobin is oxidized to methemoglobin, thereby impairing oxygen passage in the bloodstream. Furthermore, nitrite can participate in the formation of carcinogenic *N*-nitrosamines, which are strongly effective in increasing cancer risk.^3^ Therefore, a maximum permissible concentration of nitrite in drinking water is 3.0 mg L^−1^ (65 µM) according to the World Health Organization (WHO). In biological systems, the normal nitrite concentration is typically maintained within the range of 0.3–1.3 µM, whereas excessive levels may interfere with essential physiological functions, including vasodilation, mitochondrial activity, and cellular signaling pathways.^4^ Therefore, the development of accurate, reliable, and rapid analytical methods for nitrite determination is of critical importance for food safety evaluation, environmental analysis and clinical applications.^5^ In this regard, a wide range of analytical approaches has been developed for nitrite detection, including fluorescence and UV-Vis spectroscopy techniques, as well as electrochemical methods including voltammetric, potentiometric, and amperometric sensing platforms.^2^ Although these techniques generally provide high sensitivity and analytical accuracy, several technical and operational limitations remain. Conventional spectroscopic methods often require expensive instrumentation, labor-intensive procedures, and complex sample preparation protocols. Moreover, fluorescence-based assays may suffer from severe interference effects and signal instability associated with fluorescent probe systems.^6–8^

Electrochemical sensors and biosensors have emerged as powerful analytical platforms for the simple, accurate quantification and selection of single or multiple analytes in complex matrices.^9–11^ Owing to their ultrasensitive, fast response, ease of miniaturization and low cost, these sensing systems are highly recommended for portable, point-of-care, and onsite analytical applications. Consequently, electrochemical sensing platforms^12–35^ have attracted considerable attention for the direct and continuous nitrite ions monitoring in food, and biological samples. Among electrochemical techniques, amperometric and voltammetric methods are the most extensively employed due to their excellent analytical performance.^9,10^ Amperometric sensing offers high sensitivity and rapid signal response, whereas voltammetric techniques provide valuable insight into electrochemical reaction kinetics and redox mechanisms occurring at the electrode interface.^36–40^

Electrochemical determination of nitrite can be achieved either directly through the intrinsic redox activity of nitrite ions at the electrode surface or indirectly using enzymatic and labeled biosensing strategies. Non-enzymatic electrochemical sensors have gained increasing interest due to their structural simplicity, excellent operational stability, facile fabrication, and suitability for large-scale manufacturing. Such advantages enable easier quality control and improved reproducibility compared with enzyme-based platforms.^41^ Accordingly, extensive research has been devoted to non-enzymatic nitrite sensors fabrication based on a wide type of electrocatalytic materials, including metals, metal oxides, alloys, and carbon-based nanostructures, for efficient nitrite electrooxidation in complex samples.^2,11^

The successful development of high-performance non-enzymatic electrochemical sensors strongly depends on the incorporation of electrode-modifying materials that provide rapid electron-transfer kinetics, a large electrochemically active surface area, high electro–catalytic activity, long-term stability, and environmental compatibility. In this context, nanomaterials, including graphene, carbon nanotubes, metal oxides, and conducting polymers, have been extensively integrated into electrode architectures to enhance electrochemical sensing performance, particularly for the direct detection of nitrite.^42–47^

Among these nanomaterials, the unique physicochemical properties of titanium dioxide (TiO_2_) nanoparticles have attracted substantial attention, including high chemical stability, large surface area, biocompatibility, and remarkable catalytic efficiency. These characteristics significantly improve the interaction between analyte molecules and electrode surfaces, thereby enhancing sensing sensitivity and electron-transfer efficiency.^48–50^

Similarly, tungsten oxide nanostructures (WO_3_ NSs) have been widely implemented as electrocatalytic materials for nitrite electrochemical sensing, due to their low cost, high chemical stability, environmental friendliness, and relatively high specific capacitance. Moreover, nanostructured WO_3_-based sensing platforms have demonstrated excellent sensitivity, selectivity, and electrocatalytic activity toward nitrite oxidation, highlighting their potential for advanced electrochemical sensing applications.^48^

Based on these considerations, the present study focuses on the development and optimization of robust electrochemical sensing platforms for nitrite detection through the incorporation of functional bimetallic oxide nanostructures integrated with carbon nanotubes to achieve the best sensing performance. Particular emphasis is placed on investigating their sensing performance and electrocatalytic mechanisms toward nitrite oxidation, aiming to enhance analytical sensitivity, selectivity, stability, and overall sensing efficiency in complex sample matrices. By elucidating the electrochemical behavior of nitrite ions and engineering highly efficient nanostructured sensing interfaces, this work seeks to advance electrochemical nitrite sensing technologies for practical applications in food safety evaluation.

## 2. Experimental

### 2.1. Materials, instruments, and reagents

Multi-walled carbon nanotubes (MWCNTs, diameter: 10 nm, length: 3–6 µm) and graphite were brought from Sigma-Aldrich. Paraffin oil was supplied by PGi-Chemicals. Potassium hexacyanoferrate and potassium ferricyanide were purchased from Fisher Chemical and LOBA Chemie, respectively. Potassium chloride was obtained from Chem-Lab. Potassium permanganate (KMnO_4_) and hydrogen peroxide (H_2_O_2_) were supplied by ZA Chem, whereas cobalt nitrate hexahydrate (Co(NO_3_)_2_·6H_2_O), ascorbic acid, ammonium chloride, d-glucose, sodium chloride, urea, and copper sulfate pentahydrate were purchased from Piochem. Lead nitrate and sodium hydroxide were obtained from Power Chemical. Sodium acetate, glacial acetic acid, hydrochloric acid and acetic acid were also supplied by Piochem. Cobalt oxide (Co_3_O_4_), manganese oxide (MnO_2_), tungsten oxide (WO_3_), titanium dioxide (TiO_2_), and vanadium oxide (V_2_O_5_) nanostructured were purchased from a commercial supplier.

Electrochemical measurements were performed using a conventional three-electrode electrochemical system under controlled laboratory conditions. The TiO_2_–WO_3_@MWCNTs-modified carbon paste electrode (TiO_2_–WO_3_@MWCNTs/CPE) served as working electrode, while reference electrode was Ag/AgCl and counter electrode was platinum wire. All of electrochemical measurements were conducted using a PalmSens-4 potentiostat controlled by PSTrace software (version 5.11) and a Corr Test potentiostat electrochemical workstations working (CorrTest CS studio version 5.6 1102.9 software). pH meter obtained from ADWA Instruments was used for measure the pH of buffer solutions.

For preparation of acetate buffer solution (pH 4.0), sodium acetate solution adjusting with glacial acetic acid and diluted with double distilled water to the final desired volume. A solution containing ferri/ferro cyanide (5.0 mM) and KCl (0.1 M) was employed for electrochemical characterization of the different modified electrodes using electrochemical impedance spectroscopy (EIS) and cyclic voltammetry (CV) techniques.

High-resolution transmission electron microscopy (HRTEM, FEI Tecnai-T20) was used to investigate the nano-structural features and morphology of the fabricated sensing layer materials. Thermo Scientific Nicolet iS10 spectrometer used for Fourier-transform infrared (FTIR) spectroscopy measurements over spectral range of 400–4000 cm^−1^ to recognize the chemical bonding structures and characteristic the functional groups of the nanomaterials. X-ray diffraction (XRD) with Cu Kα radiation (*λ* = 1.5418 Å) over a 2*θ* scanning range of 4–80° crystallographic analysis used to determine the crystalline structure and average crystallite size of the fabricated sensing layer materials. Additionally, Raman spectroscopy measurements were performed at room temperature using a confocal Raman microscope (WITec alpha 300R) equipped with a 532 nm excitation laser operated at a laser power of 1.0 mW. Raman spectra were recorded over the wavenumber range of 100–4000 cm^−1^.

### 2.2. Preparation of TiO_2_–WO_3_@MWCNTs-modified electrode

To fabricate the carbon paste electrode (CPE), a homogeneous and uniformly textured paste was obtained by adding 1.0 g of graphite powder with 250 µL of paraffin oil (PO) in a porcelain mortar then packed into a plastic electrode holder (3.0 mm diameter) and electrically connected using a copper wire. Prior to surface modification, the electrode surface was polished on wetted Whatman filter paper to obtain a smooth and reproducible surface.

Suspensions of TiO_2_ nanoparticles, WO_3_ nanoparticles, and MWCNTs were separately prepared with a fixed concentration (5.0 mg mL^−1^), followed by ultrasonication for 20 min to ensure homogeneous dispersion. Subsequently, equal volumes of the TiO_2_, WO_3_, and MWCNTs suspensions were mixed in a volume ratio of 1 : 1 : 1 to form the TiO_2_–WO_3_@MWCNTs nanocomposite suspension. The resulting mixture was further sonicated for an additional 20 min to achieve uniform distribution of the nanocomposite constituents.

Thereafter, 5.0 µL of the prepared TiO_2_–WO_3_@MWCNTs nanocomposite suspension was drop-casted onto the polished carbon paste electrode surface and left to dry.

### 2.3. Electrochemical characterizations

For electrode characterization, CVs and EISs analysis were conducted using [Fe(CN)_6_]^3−/4−^ with a concentration of 5.0 mM mixed with 0.1 M KCl, CVs curves were performed over a scan rate of 50 mV s^−1^ and −0.8 to +1.0 V of potential window. The impedimetric spectra were carried out within 10 Hz – 10 kHz frequency range, with 10 mV AC amplitude. The EIS spectra were fitted using the Randles equivalent circuit consisting of solution resistance (*R*_s_), charge-transfer resistance (*R*_ct_), constant phase element (CPE), and Warburg impedance (*Z*_w_).

For nitrite detection, CVs curves were measured at a scan rate of 50 mV s^−1^ over −0.5 to +1.5 V potential range in acetate buffer solution (0.1 M, pH 4.0) to optimize the electrochemical response toward nitrite oxidation. Chronoamperometric measurements were subsequently conducted by applying a constant potential of +0.9 V to evaluate the sensor response, sensitivity, and current–time behavior. The limit of detection (LOD) and limit of quantification (LOQ) were calculated according to IUPAC recommendations using the slope of the linear calibration curve (*S*) and the standard deviation of replicate blank measurements (*σ*), according to the equations LOD = 3*σ*/*S* and LOQ = 10*σ*/*S*, respectively. Electrochemical measurements were performed using independently fabricated electrodes, and the results are reported as mean ± standard deviation (SD) (*n* = 3).

### 2.4. Real sample preparation and analysis

Food samples, including burger, sausage, and salami, were collected from local markets in Egypt. Approximately the homogenized, dried and grinded food samples (5.0 g) were transferred into 50 mL of acetate buffer solution (0.1 M, pH 4.0). The mixture was thoroughly stirred and sonicated at 75 °C for 25 min to ensure efficient extraction of nitrite ions. After extraction, the samples were filtered through Whatman filter paper to remove suspended solid residues. The resulting filtrates were used directly for electrochemical nitrite determination without further treatment. For analysis, 500 µL of each filtered sample was diluted with 4500 µL of acetate buffer (pH 4.0), and the resulting solution was subjected to chronoamperometric measurement at +0.9 V for the quantitative determination of nitrite ions.

## 3. Results and discussion

### 3.1. Morphological and functional characterization

**Fig. 1:**
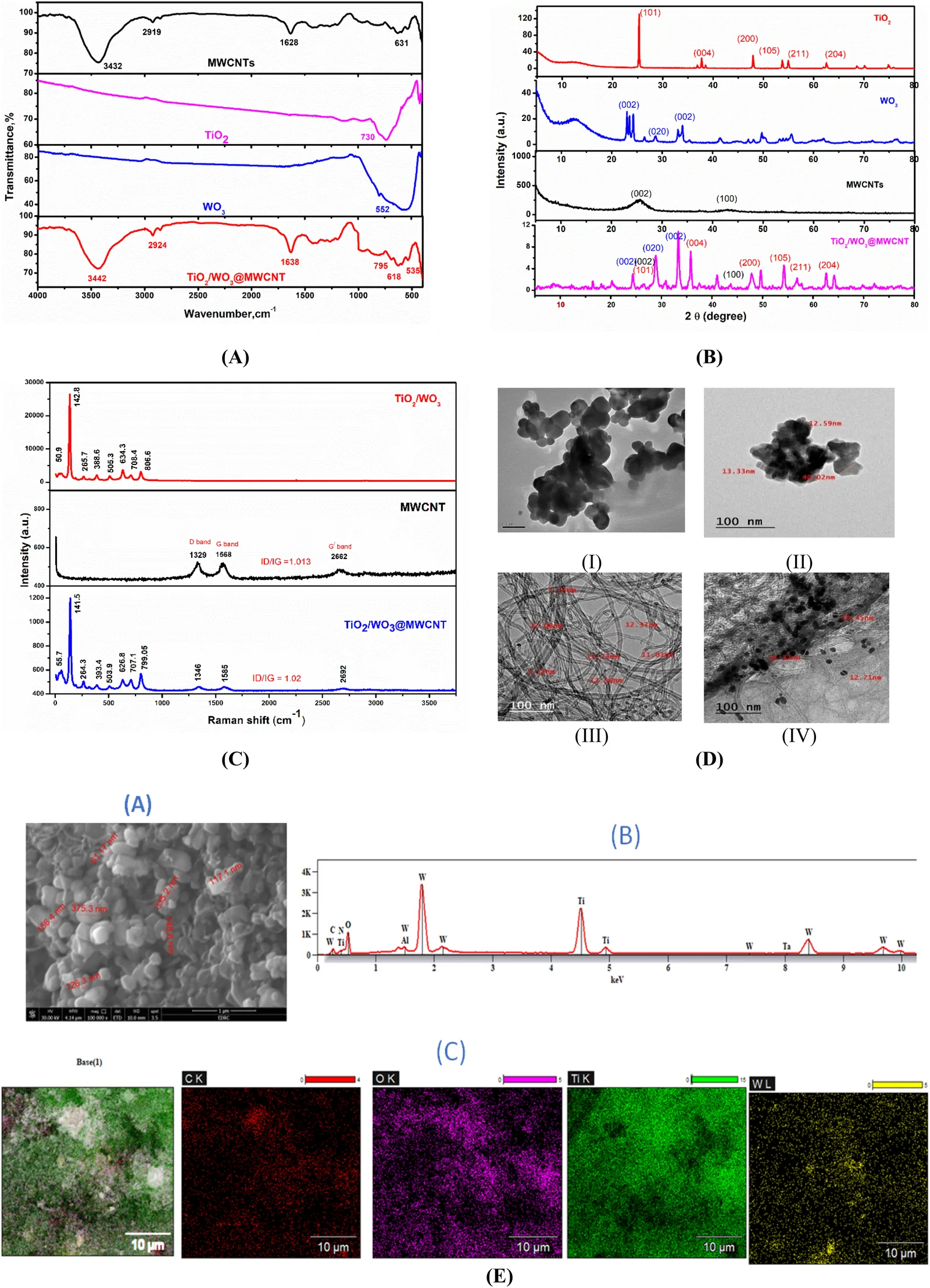
(A) Infrared spectra, (B) XRD patterns of MWCNTs, WO_3_, TiO_2_, and TiO_2_–WO_3_@MWCNTs nanocomposites. (C) Raman spectra of TiO_2_/WO_3_, MWCNTs, and TiO_2_–WO_3_@MWCNTs nanocomposite, illustrating the characteristic vibrational modes and the shifts of the Ti–O and W–O bands resulting from interfacial interactions. (D) TEM images of (I) TiO_2_, (II) WO_3_, (III) MWCNTs, and (IV) the TiO_2_–WO_3_@MWCNTs nanocomposite, illustrating their morphological features and nanoscale architecture. (E): Morphological and elemental characterization of the TiO_2_–WO_3_@MWCNTs nanocomposite. (A) SEM micrographs of the TiO_2_–WO_3_@MWCNTs nanocomposite, revealing the successful decoration of TiO_2_ and WO_3_ nanostructures on the interconnected MWCNTs network. (B) EDX spectrum confirming the elemental composition of the nanocomposite. (C) Elemental mapping images showing the spatial distribution of C, O, Ti, and W, demonstrating the homogeneous dispersion of TiO_2_ and WO_3_ nanoparticles throughout the MWCNTs framework, indicating the successful formation of the TiO_2_–WO_3_@MWCNT nanocomposite.

To identify the characteristic structural and functional features of the nanostructured electrode modifiers, FTIR spectroscopy was carried out for TiO_2_, WO_3_, MWCNTs, and the TiO_2_–WO_3_@MWCNTs nanocomposite, as presented in Fig. 1A.

The FTIR spectrum of TiO_2_ exhibited broad absorption bands in the range of 400–900 cm^−1^, owing to Ti–O–Ti and Ti–O stretching vibrations, which are characteristic of the anatase phase. Additional bands observed at approximately 3400 cm^−1^ and 1620 cm^−1^ are attributed to O–H stretching and H–O–H bending vibrations, respectively, indicating surface-adsorbed water molecules and hydroxyl functional groups. Similarly, WO_3_ showed prominent metal–oxygen vibrational bands within 500–900 cm^−1^, with a distinct peak near 750 cm^−1^ and an additional band around 960 cm^−1^ assigned to terminal W=O stretching vibrations, particularly associated with hydrated tungsten oxide species.

For functionalized MWCNTs, characteristic absorption bands are observed for C=O stretching at 1721 cm^−1^, C=C stretching at 1620 cm^−1^, and C–O stretching at 1100–1200 cm^−1^, along with a broad band at 3400 cm^−1^ for O–H stretching. Notably, in the FTIR spectrum of the TiO_2_–WO_3_@MWCNTs nanocomposite, a reduction and partial attenuation of the characteristic vibrational bands of TiO_2_ and WO_3_ is observed, indicating strong interfacial interactions and effective metal oxide nanostructures integration with the carbon nanotube network.

In addition to the FTIR analysis, X-ray diffraction (XRD) analysis (Fig. 1B) further confirms the crystalline nature and phase composition of the prepared materials. TiO_2_ exhibits well-defined diffraction peaks were recorded at 2*θ* = 62.6°,55.2°, 53.8°,48.1°, 37.7°, and 25.2°, indexed to the (204), (211), (105), (200), (004), and (101) planes of the anatase phase (JCPDS No. 21-1272), confirming a highly crystalline tetragonal structure.

WO_3_ provided characteristic reflections at 2*θ* = 23.1°, 23.6°, 24.4°, 33.4°, and 34.1°, corresponding to the monoclinic phase (JCPDS No. 43-1035), proofed its crystalline framework. MWCNTs exhibited a wide peak at 2*θ* = 25.5–26.0°, indexed to the (002) graphitic plane, along with a weaker reflection around 42–43°, attributed to the graphitic carbon of (100) plane. These XRD diffraction patterns confirmed the coexistence of crystalline metal oxides and graphitic carbon within the composite structure.

Furthermore, Raman spectroscopy was employed to investigate the vibrational characteristics and structural features of the prepared TiO_2_–WO_3_@MWCNTs nanocomposite. The Raman spectra of the individual TiO_2_, WO_3_, and MWCNT components were compared with that of the resulting composite, as shown in Fig. 1C. The Raman spectrum of TiO_2_ exhibited a characteristic band at approximately 142.6 cm^−1^, which can be assigned to the vibrational mode of anatase TiO_2_. After incorporation of WO_3_ and MWCNTs, this band shifted slightly to approximately 141.5 cm^−1^. Such a shift indicates a change in the local vibrational environment of TiO_2_ and may be associated with lattice strain, interfacial interactions, or changes in the local bonding environment resulting from composite formation. Subsequently, the Raman spectrum of WO_3_ exhibited a characteristic band at approximately 806.6 cm^−1^, attributable to W–O stretching vibrations. In the TiO_2_–WO_3_@MWCNTs composite, this band shifted to approximately 799.0 cm^−1^, indicating a modification of the local vibrational environment of the WO_3_ phase. The simultaneous shifts observed for the TiO_2_ and WO_3_ bands are consistent with structural and interfacial interactions following formation of the composite.

The characteristic Raman bands of MWCNTs were also retained in the composite. The D band, observed at approximately 1329 cm^−1^, is associated primarily with disorder and defect-related scattering in the carbon framework, whereas the G band at approximately 1568 cm^−1^ originates from the in-plane vibrational mode of sp^2^-hybridized carbon. The presence of both bands confirms the retention of the characteristic graphitic structure of the MWCNTs after incorporation of TiO_2_ and WO_3_. The *I*_D_/*I*_G_ ratio, representing the relative intensity of the defect-related D band to the graphitic G band, was used to evaluate the degree of structural disorder in the MWCNTs. Whereas, the *I*_D_/*I*_G_ ratio increased slightly from 1.013 for pristine MWCNTs to 1.020 for the TiO_2_–WO_3_@MWCNTs composite, indicating a small increase in defect-related disorder following modification. Nevertheless, the minimal change in this ratio suggested that the overall graphitic framework of the MWCNTs was largely preserved after the composite formation.

For further characterization, size, and shape of the materials were identified using the 2D-TEM images (Fig. 1D), which provided detailed insight into the morphology and nanoscale architecture of the fabricated sensing layer materials. In this regard, The TiO_2_ powder materials showed spherical size distribution with a range of 10–25 nm. Clear lattice fringes are observed with an interplanar spacing of 0.35 nm, indexed to the (101) planes of anatase TiO_2_, confirming high crystallinity. However, the WO_3_ particles appeared as slightly larger agglomerates with particle sizes between 20 and 50 nm, while their observed lattice spacing of 0.37 nm is attributed to the monoclinic plane of WO_3_ (002).

MWCNTs exhibit well-defined hollow tubular structures composed of multiple concentric graphitic layers, with outer diameters of 10–30 nm and lengths extending to several micrometers. The measured interlayer spacing of 0.34 nm matches with the (002) planes of graphitic carbon. In the nanocomposite, TiO_2_ and WO_3_ nanoparticles are uniformly anchored onto the external walls of MWCNTs, indicating strong interfacial coupling. This hierarchical architecture enhances electron transport pathways and increase the electroactive sites number.

The combined FTIR, XRD, and TEM analyses confirm the successful fabrication of a well-integrated TiO_2_–WO_3_@MWCNTs nanocomposite, which provides synergistic structural and electronic advantages that significantly enhance electrocatalytic activity and electron-transfer kinetics toward nitrite oxidation.

The morphology and elemental composition of the TiO_2_–WO_3_@MWCNTs nanocomposite were investigated using scanning electron microscopy (SEM) coupled with energy-dispersive X-ray spectroscopy (EDX) and elemental mapping analysis (see Fig. 1D(I–III)). The SEM images revealed the successful formation of a three-dimensional interconnected nanostructure, where TiO_2_ and WO_3_ nanostructures were uniformly anchored onto the surface of the multi-walled carbon nanotubes (MWCNTs). The MWCNTs formed a conductive network that effectively prevented the aggregation of the metal oxide nanoparticles, resulting in a rough and porous surface with abundant active sites. Such a hierarchical architecture is expected to facilitate electrolyte diffusion, accelerate electron transfer, and provide a large electrochemically active surface area, thereby enhancing the sensing performance of the fabricated electrode. The EDX spectrum confirmed the presence of titanium (Ti), tungsten (W), oxygen (O), and carbon (C) as the principal elements of the nanocomposite, with no detectable impurity peaks, demonstrating the high purity and successful synthesis of the TiO_2_–WO_3_@MWCNTs material. Furthermore, the elemental mapping images exhibited a homogeneous spatial distribution of Ti, W, O, and C throughout the nanocomposite, confirming the uniform decoration of TiO_2_ and WO_3_ on the MWCNTs framework. The intimate contact between the metal oxides and the conductive carbon nanotubes is anticipated to promote efficient charge transport and synergistic interactions, contributing to the enhanced electrocatalytic activity and stability of the TiO_2_–WO_3_@MWCNTs-modified electrode.

### 3.2. Electrochemical characterizations

**Fig. 2:**
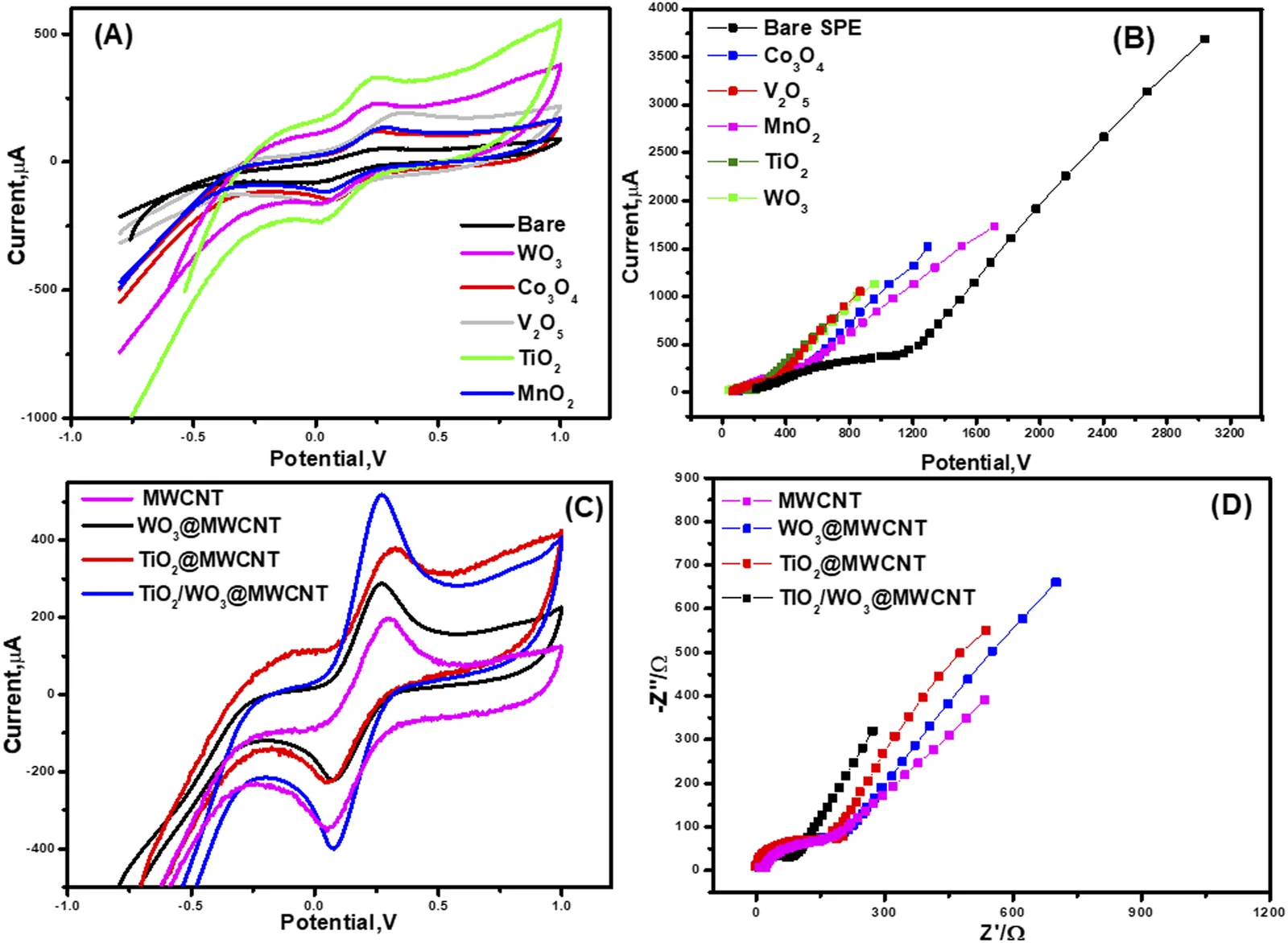
(A) Cyclic voltammograms (CVs) and (B) electrochemical impedance spectroscopy (EIS) Nyquist plots of CPEs modified with different metal oxide nanostructures (MnO_2_, Co_3_O_4_, WO_3_, TiO_2_, and V_2_O_5_). (C) CVs and (D) EIS Nyquist plots of MWCNTs/CPE, WO_3_@MWCNTs/CPE, TiO_2_@MWCNTs/CPE, and TiO_2_–WO_3_@MWCNTs/CPE. All electrochemical measurements were performed in 5.0 mM [Fe(CN)_6_]^3−/4−^ containing 0.1 M KCl at a scan rate of 50 mV s^−1^.

To develop an efficient electrochemical sensing platform for the sensitive and fast detection of trace nitrite ions in food samples, several nanostructured materials were electrochemically screened using both CV and EIS, respectively. The objective of this screening process was to evaluate the electrochemically active surface area, investigate the electrocatalytic behavior of the nanomaterials, and assess their capability to facilitate the transfer of electron between the redox species and the electrode interface.^35,36^ Accordingly, the proposed nanomaterials were electrochemically characterized using either a standard redox probe of 5.0 mM ferri/ferrocyanide ([Fe(CN)_6_]^3−/4−^) or nitrite ions (NO_2_^−^) as the target analyte. The primary selection criteria were based on the voltammetric redox behavior of the ferri/ferrocyanide system, the corresponding EIS responses, and the enhancement in nitrite oxidation current at the modified carbon paste electrodes (CPEs). As illustrated in Fig. 2a, all metal oxide-modified electrodes, including MnO_2_, V_2_O_5_, Co_3_O_4_, WO_3_, and TiO_2_, exhibited significantly enhanced redox currents compare to the unmodified CPE. The oxidation peak current (*I*_pa_) for the modified electrodes reached approximately 300 µA, whereas the unmodified CPE displayed a considerably lower current response of less than 60 µA. Among the investigated materials, TiO_2_ and WO_3_-modified electrodes demonstrated the highest voltammetric response toward the ferri/ferrocyanide redox probe (see Table 1), indicating superior electrochemical activity and enhanced electron-transfer capability. The EIS measurements represented by Nyquist plots (Fig. 2b) and the equivalent electric circuit which used for fitting the EIS data confirmed the enhanced electrochemical characteristics of the nanostructured electrodes. The impedance spectra exhibited dominant Warburg diffusion behavior, evidenced by the pronounced linear regions extending along both the real and imaginary impedance axes. This behavior indicates that diffusion-controlled electrochemical processes predominated at the electrode interface, which is characteristic of highly porous and nanostructured materials. Moreover, the charge-transfer resistance (*R*_ct_) values were significantly reduced upon electrode modification. The bare electrode exhibited an *R*_ct_ value of 1300 Ω, whereas MnO_2_^−^, V_2_O_5_^−^, Co_3_O_4_^−^, WO_3_^−^, and TiO_2_-modified electrodes displayed reduced *R*_ct_ values of approximately 620, 470, 680, 420, and 240 Ω, respectively (see Table 1),. These findings are matched with the CVs results and confirm the enhanced electron-transfer kinetics induced by the nanomaterial modification. Based on the obtained electrochemical performance, TiO_2_ and WO_3_ were selected as the primary electrocatalytic components for further integration with the MWCNTs to improve both electrical conductivity and catalytic activity. The resulting TiO_2_–WO_3_@MWCNTs-modified CPE exhibited a remarkable enhancement in nitrite oxidation current, increasing from 58 µA at the bare electrode to 519 µA at the composite-modified electrode, corresponding to an approximately nine-fold increase in electrochemical response (Fig. 2c). Although TiO_2_-modified electrodes demonstrated catalytic activity toward nitrite oxidation, EIS analysis (Fig. 2d) revealed relatively high charge-transfer resistance values (240 Ω), which may be attributed to nanoparticle agglomeration and limited electrical conductivity. In contrast, TiO_2_@MWCNTs modified electrode exhibited substantially lower charge-transfer resistance (200 Ω) due to the superior electrical conductivity and interconnected conductive network provided by the carbon nanotubes. Notably, the incorporation of both TiO_2_ and WO_3_ into the MWCNTs framework produced a synergistic enhancement in electrochemical performance. The TiO_2_–WO_3_@MWCNTs composite exhibited the lowest charge-transfer resistance (150 Ω), indicating highly accelerated electron-transfer kinetics (see Table 1). This improvement can be attributed to the synergistic interaction between the crystalline TiO_2_–WO_3_ nanoparticles and the conductive tubular structure of MWCNTs, which collectively provide abundant electroactive sites, efficient electron transport pathways, and enhanced nitrite adsorption capability, thereby significantly improving the electrocatalytic oxidation efficiency of nitrite ions.

**Table 1:** Summary of the electrochemical parameters obtained from Fig. 2.^a^

Electrode type	*I*_a_ (µA)	*I*_c_ (µA)	*E*_oxd._ (V)	*E*_red._ (V)	Δ*E*_p_	*R*_s_ (Ω)	*R*_ct(1)_ (Ω)	CPE *n* (µF)	*W* (Ω)
Bare (unmodified CE)	58.8	−79.2	0.27	0.02	0.25	186	1300	0.125 0.896	314
Co_3_O_4_	128	−157	0.25	0.01	0.24	110	680	0.184 0.921	348
V_2_O_5_	200	−174	0.35	0.01	0.34	54	470	0.248 0.854	372
WO_3_	237	−166	0.25	0.05	0.20	27	420	0.249 0.954	553
TiO_2_	338	−246	0.24	0.01	0.23	71	240	0.258 0.912	575
MnO_2_	144	−124	0.27	0.04	0.23	77	620	0.192 0.894	351
MWCNTs	200	−351	0.30	0.05	0.25	279	270	0.255 0.861	243
WO_3_@MWCNTs	294	−225	0.26	0.06	0.20	279	280	0.254 0.912	582
TiO_2_@MWCNTs	380	−233	0.31	0.04	0.27	263	200	0.259 0.896	561
TiO_2_–WO_3_@MWCNTs	520	−401	0.26	0.07	0.19	281	110	0.261 0.835	599

a The current of anodic peak (*I*_a_); the current of cathodic peak (*I*_c_); constant phase element (CPE); potential of oxidation (*E*_ox_), potential of reduction (*E*_red_); resistance of solution (*R*_s_), charge-transfer resistance (*R*_ct_) and Warburg resistance (*W*).

**Fig. 3:**
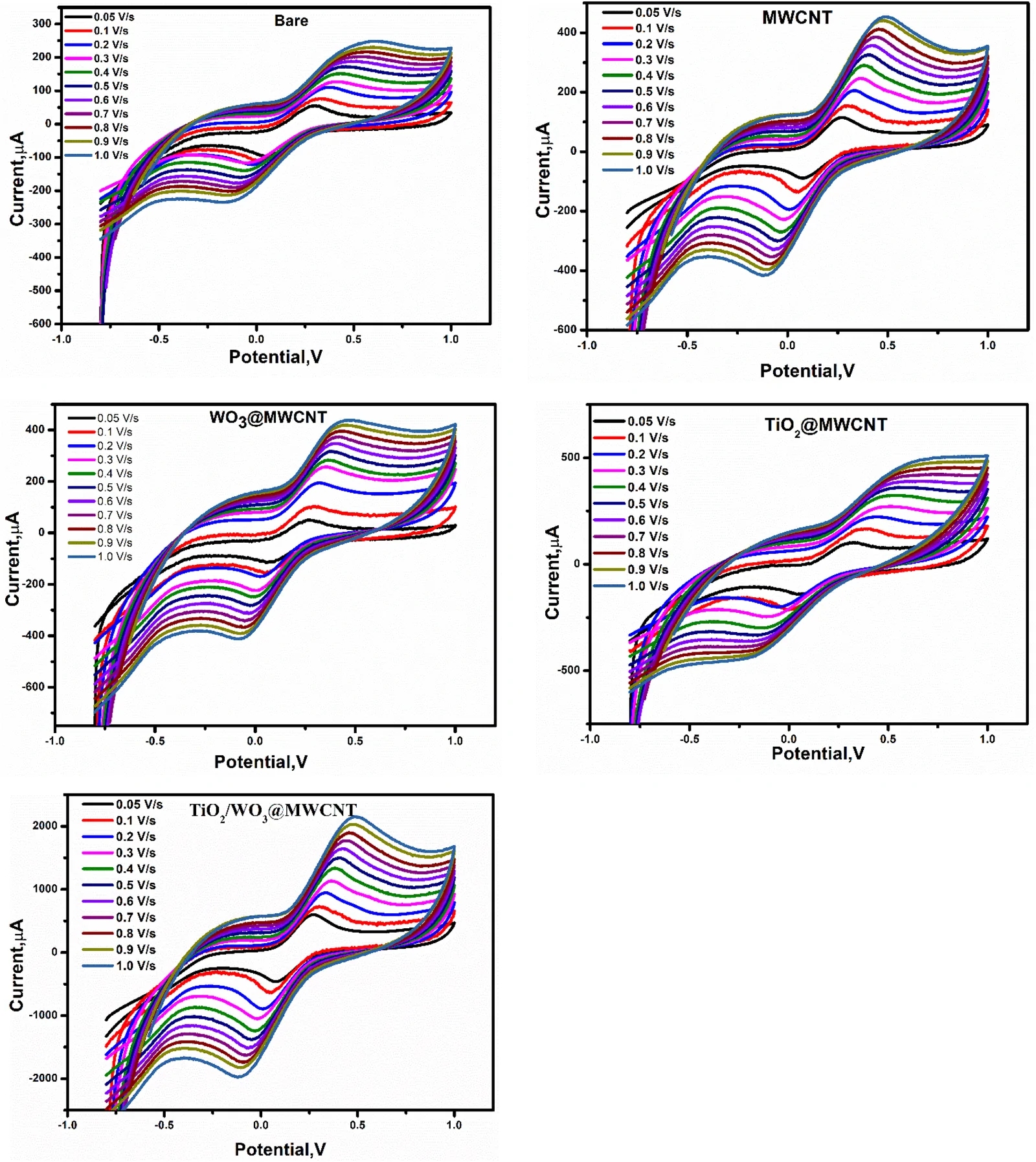
Effect of scan rate variation (from 0.05 to 1.0 V s^−1^) on the cyclic voltammetric responses of bare-CPE, MWCNTs/CPE, WO_3_@MWCNTs/CPE, TiO_2_@MWCNTs/CPE, and TiO_2_–WO_3_@MWCNTs/CPE modified electrodes. Electrochemical measurements were performed in a ferri/ferrocyanide redox probe solution to evaluate the electron-transfer characteristics and electroactive surface behavior of the fabricated electrodes.

**Fig. 4:**
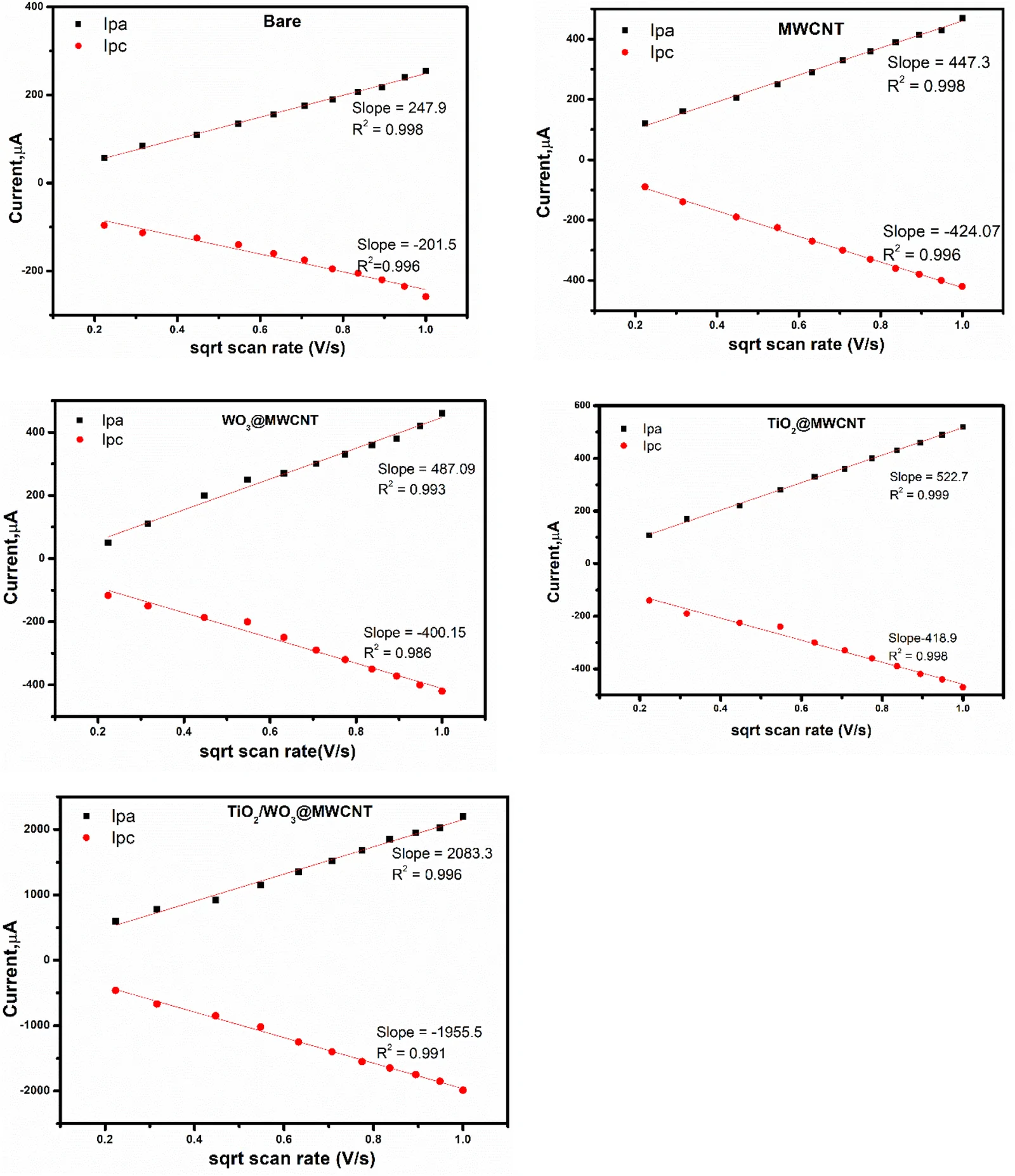
The corresponding calibration plot of the scan rate^1/2^*vs.* the peak current.

Consequently, CV measurements were performed at different scan rates (10 to 100 mV s^−1^) using TiO_2_–WO_3_@MWCNTs-modified electrode, as illustrated in Fig. 3 to estimate the electroactive surface area of the fabricated electrodes. In this regard, the current of anodic peak (*I*_pa_) increased with increasing scan rate, indicate enhancing in electron-transfer activity at the electrode interface. A linearity relationship between the current of oxidation peak and the square root of the scan rate (*ν*^1/2^) was observed as presented in Fig. 4, following the regression equation:*I*_pa_ (mA) = 319.6396*v*^1/2^ – 676.3417 (*R*^2^ = 0.998)

The excellent linearity confirmed that the electrochemical redox process occurring at the TiO_2_–WO_3_@MWCNTs is predominantly diffusion-controlled.

Further, to evaluate the electroactive surface area of the fabricated electrodes, the Randles–Sevcik equation was employed:*I*_pa_ = (2.69 × 10^5^)*n*^3/2^CAD^1/2^*v*^1/2^where *ν* is the scan rate (V s^−1^), *D* is the diffusion coefficient (cm^2^ s^−1^), *C* is the concentration of the redox species (mol cm^−3^), *A* is the electroactive surface area (cm^2^), *n* is the number of electrons transferred, and *I*_pa_ is the anodic peak current (A).

Using the above equation, the electrochemically active surface areas of the fabricated electrodes were calculated. The unmodified electrode exhibited an electrochemically active surface area of approximately 0.066 cm^2^, whereas the MWCNTs-, WO_3_@MWCNTs-, and TiO_2_@MWCNTs-modified electrodes showed increased values of approximately 0.120, 0.126, and 0.140 cm^2^, respectively. Notably, the TiO_2_–WO_3_@MWCNTs-modified electrode exhibited a substantially larger electrochemically active surface area of approximately 0.556 cm^2^, corresponding to an approximately 8.4-fold increase compared with the unmodified electrode.

The increase in electrochemically active surface area may be attributed to the synergistic integration of TiO_2_ and WO_3_ nanoparticles within the highly conductive and porous MWCNTs network, providing additional electroactive sites and facilitating efficient electron transport.

The electrochemical reversibility of the electrode was quantitatively investigated by monitoring the peak-to-peak potential separation (Δ*E*_p_) of the ferri/ferrocyanide redox couple at different scan rates for TiO_2_–WO_3_@MWCNTs modified electrode. The separation was determined from the difference between the anodic and cathodic peak potentials, according to Δ*E*_p_ = ∣*E*_pa_ − *E*_pc_∣. As summarized in Table S1, the Δ*E*_p_ value increased from 197.0 mV at 0.05 V s^−1^ to 623.4 mV at 1.0 V s^−1^. A moderate increase was observed at lower scan rates, with Δ*E*_p_ reaching approximately 347 mV at 0.4 V s^−1^, followed by a more pronounced increase at higher scan rates. The progressive enlargement of the peak separation with increasing scan rate indicates increasing electrode polarization and suggests that electron-transfer kinetics contribute to the overall electrochemical response. These findings are characteristic of a quasi-reversible electron-transfer process, in which the electron-transfer rate becomes increasingly influential as the scan rate increases. Thus, the Δ*E*_p_ analysis provides quantitative support for the electrochemical behavior of the modified electrode and complements the cyclic voltammetry results.

#### 3.2.1 Effect of scan rate and estimation of the number of transferred electrons and diffusion coefficient value

**Fig. 5:**
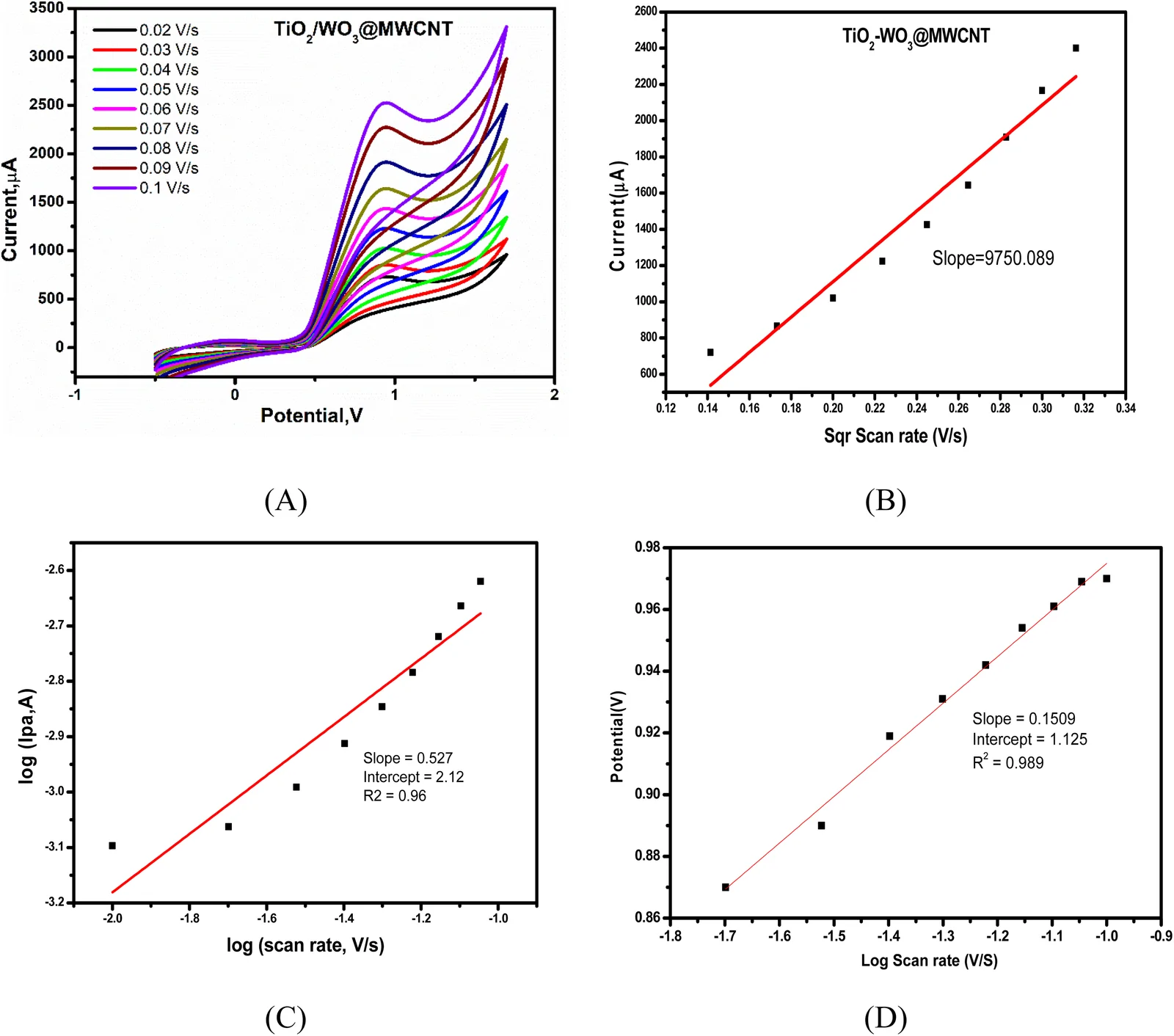
(A) Cyclic voltammograms of the TiO_2_–WO_3_@MWCNTs-modified electrode in 0.1 M acetate buffer (pH 4.0) containing 30 mM nitrite at different scan rates (0.02–0.10 V s^−1^). (B) Linear relationship between the oxidation peak current and the square root of the scan rate. (C) Dependence of the oxidation peak potential on the logarithm of the scan rate, indicating scan-rate-dependent electrochemical kinetics. (D) The anodic peak potential (*E*_p_) *vs.* the logarithm of the scan rate (log *v*).

The influence of scan rate on the electrochemical oxidation of nitrite at the TiO_2_–WO_3_@MWCNT/CPE electrode was investigated to elucidate the controlling mechanism of the electrode reaction. Cyclic voltammograms were recorded at scan rates ranging from 20 to 100 mV s^−1^, while the nitrite concentration and other experimental conditions were maintained constant. As illustrated in Fig. 5A, the oxidation peak current progressively increased as the scan rate increased, indicating a faster electrochemical response at higher scan rates. To determine whether the oxidation process was controlled predominantly by diffusion or surface adsorption, the anodic peak current (*I*_pa_) was plotted against the square root of the scan rate (*v*^1/2^). A good linear correlation was obtained between *I*_pa_ and *v*^1/2^ (Fig. 5B), demonstrating that the oxidation of nitrite at the TiO_2_–WO_3_@MWCNT/CPE electrode is mainly diffusion controlled rather than governed by surface adsorption. This behavior was further supported by the linear log (*I*_p_) *versus* log (*v*) relationship, which exhibited a slope of approximately 0.52 (Fig. 5C), close to the theoretical value of 0.5 expected for a diffusion-controlled process.

In addition, a gradual displacement of the oxidation peak potential toward more positive values was observed as the scan rate increased. The dependence of the anodic peak potential (*E*_p_) on the logarithm of the scan rate (log *v*) was therefore examined (Fig. 5D). A linear relationship was obtained and described by the following equation: *E*_p_ (V) = 0.1509 log *v* + 1.125 with: *R*^2^ = 0.989. The linear dependence of *E*_p_ on log *v* indicates that the nitrite oxidation process possesses an irreversible or kinetically limited electron-transfer character. The number of electrons involved in the oxidation process was further evaluated using the Laviron model. For an irreversible electrode reaction, the peak potential can be expressed as:
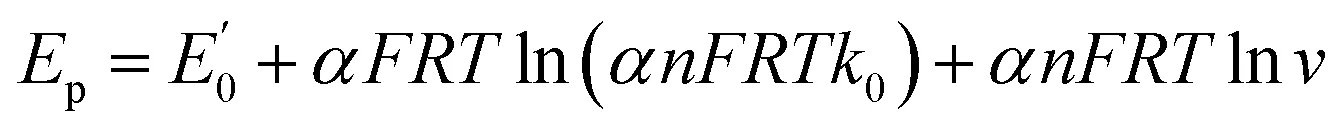
where 
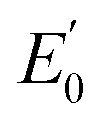
 is the formal potential, *R* is the gas constant (8.314 J mol^−1^ K^−1^), *T* is the absolute temperature (298.15 K), *α* is the electron–transfer coefficient, *n* is the number of electrons transferred, *F* is the Faraday constant (96 485 C mol^−1^), and *k*_0_ is the standard heterogeneous electron-transfer rate constant. From the slope of the *E*_p_*versus* log *v* plot, the value of *αn* was estimated to be 0.1509.

The electron–transfer coefficient was subsequently estimated from the difference between the peak potential and the potential corresponding to half of the peak current. The following relationship was employed:*E*_p_ − *E*_p1/2_ = *αnF*1.857*RT*where *E*_p1/2_ represents the potential at which the current reaches half of the peak current. The calculated value of *α* was approximately 0.076. Combining this value with the experimentally obtained *αn* value gave an estimated electron-transfer number of approximately 1.98, which can reasonably be approximated to 2 electrons. This result supports the involvement of a two-electron oxidation pathway in the electrochemical conversion of nitrite at the TiO_2_–WO_3_@MWCNT/CPE electrode. Accordingly, the proposed overall oxidation reaction can be represented as:NO_2_^−^ + H_2_O → NO_3_^−^ + 2H^+^ + 2e^−^

The obtained kinetic results therefore indicate that nitrite oxidation at the TiO_2_–WO_3_@MWCNT/CPE electrode is predominantly diffusion controlled and involves approximately two electrons, while the dependence of *E*_p_ on log *v* indicates an irreversible electron-transfer process. The improved response can be attributed to the highly conductive MWCNT network and the TiO_2_/WO_3_ heterointerface, which facilitate electron transport and promote nitrite oxidation at the modified electrode surface. Because the nitrite oxidation exhibited irreversible behavior, the diffusion coefficient was estimated using the irreversible Randles–Ševčík equation. The electron-transfer number was approximated as two based on the proposed two-electron oxidation of nitrite to nitrate, while the electron–transfer coefficient was obtained from the Laviron analysis. The diffusion coefficient of nitrite was further estimated from the scan-rate-dependent voltammetric response using the irreversible Randles–Ševčík equation. Since the oxidation of nitrite exhibited an irreversible electrochemical character, the relationship *I*_p_ = 2.99 × 10^5^*n*(*αn*)^1/2^*ACD*^1/2^*v*^1/2^ was employed, where *I*_p_ is the oxidation peak current, *n* is the number of transferred electrons, *α* is the electron-transfer coefficient, *A* is the electroactive surface area, *C* is the nitrite concentration, *D* is the diffusion coefficient, and *v* is the scan rate. Using *n* = 1.98, *α* = 0.076, *A* = 0.140 cm^2^, *C* = 30 mM, and a slope of 9750 µA (V s^−1^)^−1/2^ obtained from the *I*_p_*versus v*^1/2^ plot, the diffusion coefficient was estimated to be 1.87 × 10^−4^ cm^2^ s^−1^. This result supports the efficient mass transport of nitrite toward the TiO_2_–WO_3_@MWCNT-modified electrode surface.

### 3.3. Electrochemical assay optimization

**Fig. 6:**
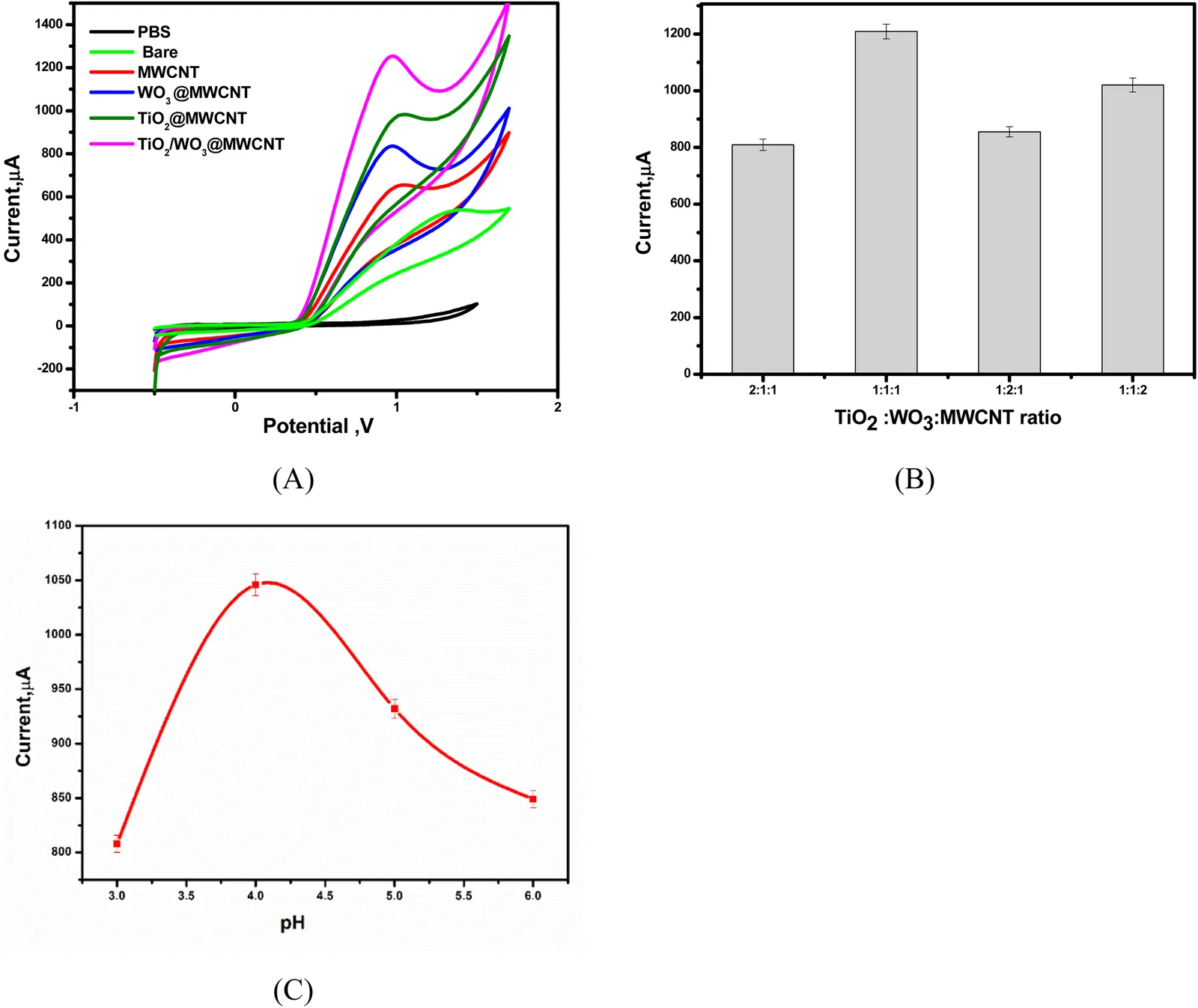
(A) CVs of MWCNTs, TiO_2_@MWCNTs, WO_3_@MWCNTs, and TiO_2_–WO_3_@MWCNTs-modified CPEs recorded in the presence of a fixed concentration of nitrite (30 mM) with a scan rate of 50 mV s^−1^, illustrating the comparative electrocatalytic performance toward nitrite oxidation. (B) Effect of the TiO_2_ : WO_3_ : MWCNTs composition ratio on the electrochemical response toward nitrite under identical experimental conditions. (C) The influence of solution pH on nitrite oxidation was subsequently examined using 0.1 M acetate buffer over the pH range of 3.0–6.0.

CV comparison at 40 mM nitrite demonstrated that the TiO_2_–WO_3_@MWCNTs-modified CPE exhibited the highest oxidation current among the investigated electrodes (Fig. 6A). Accordingly, TiO_2_–WO_3_@MWCNTs was selected as the optimal electrode modifier, and the experimental parameters affecting nitrite detection were systematically optimized.

First, the effect of the relative composition of TiO_2_, WO_3_, and MWCNTs was investigated. Nanocomposites with TiO_2_ : WO_3_ : MWCNTs ratios of 2 : 1 : 1, 1 : 1 : 1, 1 : 2 : 1, and 1 : 1 : 2 were prepared and evaluated under identical electrochemical conditions. As shown in Fig. 6B, the 1 : 1 : 1 composition produced the highest nitrite oxidation current, indicating the most favorable electrochemical performance among the investigated formulations. This enhanced response is attributed to the balanced integration of the electroactive TiO_2_ and WO_3_ components with the highly conductive MWCNT network. Increasing the relative proportion of either oxide may partially compromise the conductive pathways, whereas excessive MWCNTs content may reduce the density of accessible oxide-based catalytic sites. Therefore, the 1 : 1 : 1 TiO_2_ : WO_3_ : MWCNTs ratio was selected as the optimized composition for subsequent measurements.

Second, the influence of solution pH on nitrite oxidation was subsequently examined using 0.1 M acetate buffer over the pH range of 3.0–6.0. As shown in Fig. 6C, the oxidation current increased with increasing pH and reached a maximum at pH 4.0, followed by a gradual decrease at higher pH values. The pronounced pH dependence can be associated with changes in nitrite speciation, surface protonation, and proton-assisted interfacial electron–transfer processes. Under mildly acidic conditions, favorable interactions between nitrite species and the protonated metal-oxide surface may facilitate interfacial electron transfer and enhance nitrite oxidation. At higher pH values, changes in the surface protonation state and interfacial charge can adversely affect nitrite adsorption and electron-transfer kinetics. Accordingly, pH 4.0 was selected as the optimum working condition, providing the highest and most stable voltammetric response.

The analytical performance of the TiO_2_–WO_3_@MWCNTs-modified electrode was subsequently evaluated by cyclic voltammetry using different nitrite concentrations. The electrode exhibited a clear and concentration-dependent increase in the nitrite oxidation peak current, demonstrating its effective electrocatalytic response toward nitrite.

**Fig. 7:**
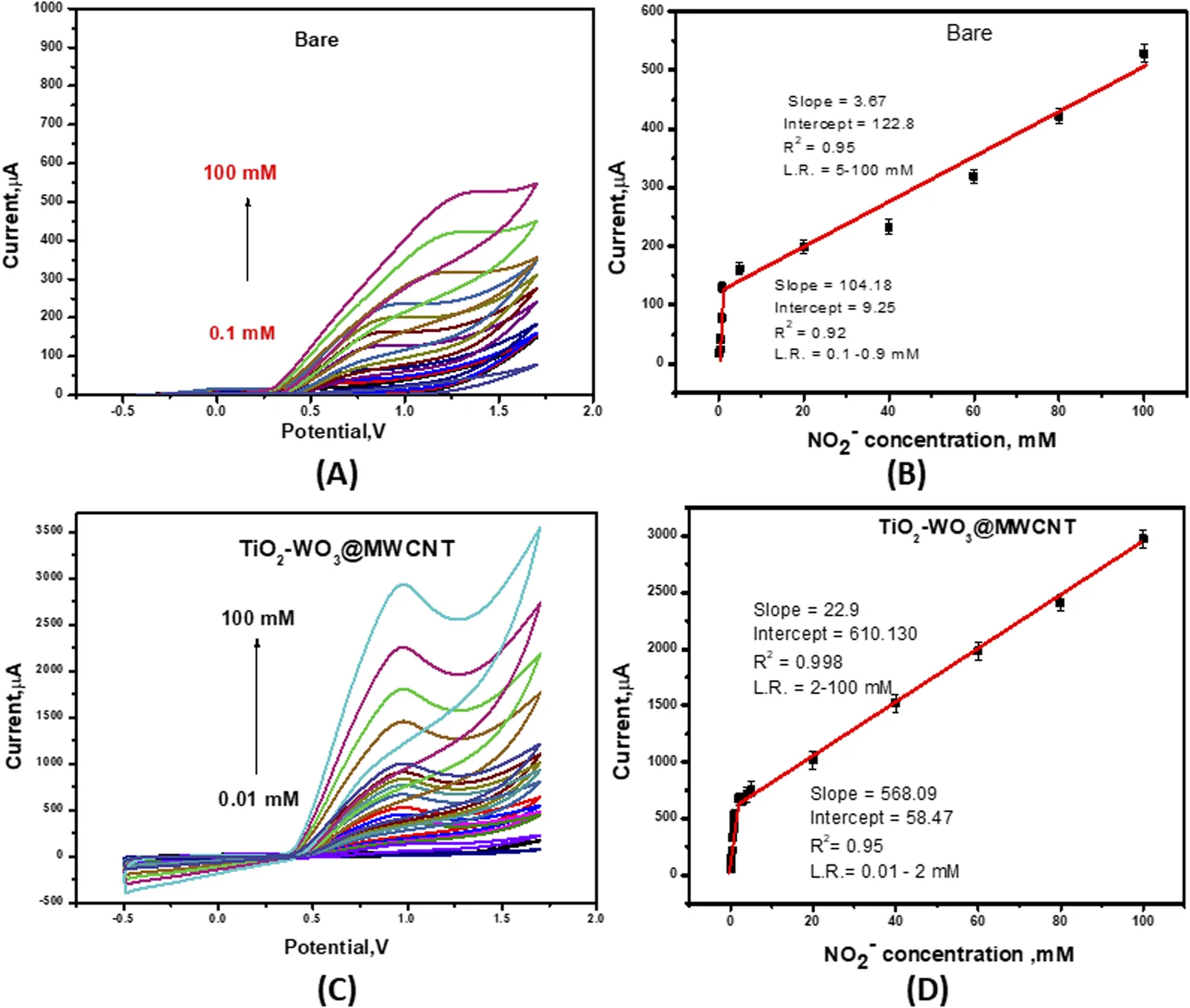
(A) CV responses and (B) linear calibration plot of oxidation peak current *vs.* nitrite concentration of bare CPE. (C) CV responses and (D) linear calibration plot of oxidation peak current *vs.* nitrite concentration of TiO_2_–WO_3_@MWCNTs/CPE. Using different concentrations of nitrite ions (0.01–100 mM) at 50 mV s^−1^ scan rate.

As shown in Fig. 7A–D, the oxidation peak current increased linearly with nitrite concentration over the range of 0.01–100 mM. The TiO_2_–WO_3_@MWCNTs/CPE exhibited a sensitivity of 22.9 µA mM^−1^ with excellent linearity (*R*^2^ = 0.999) and a detection limit of 0.003 mM. In comparison, the unmodified electrode showed substantially lower sensitivity (3.67 µA mM^−1^) and a narrower linear range of 0.1–100 mM. Thus, incorporation of the TiO_2_–WO_3_@MWCNTs nanocomposite markedly enhanced the electrochemical response toward nitrite. The superior sensing performance can be attributed to the synergistic combination of the highly conductive MWCNT network and the electroactive TiO_2_ and WO_3_ components. This hybrid architecture facilitates efficient electron transport, increases the electrochemically accessible surface area, and provides abundant active sites for nitrite adsorption and oxidation, collectively leading to enhanced electrocatalytic activity and improved analytical sensitivity.

#### 3.3.1 Chronoamperometric analysis and calibration curve

**Fig. 8:**
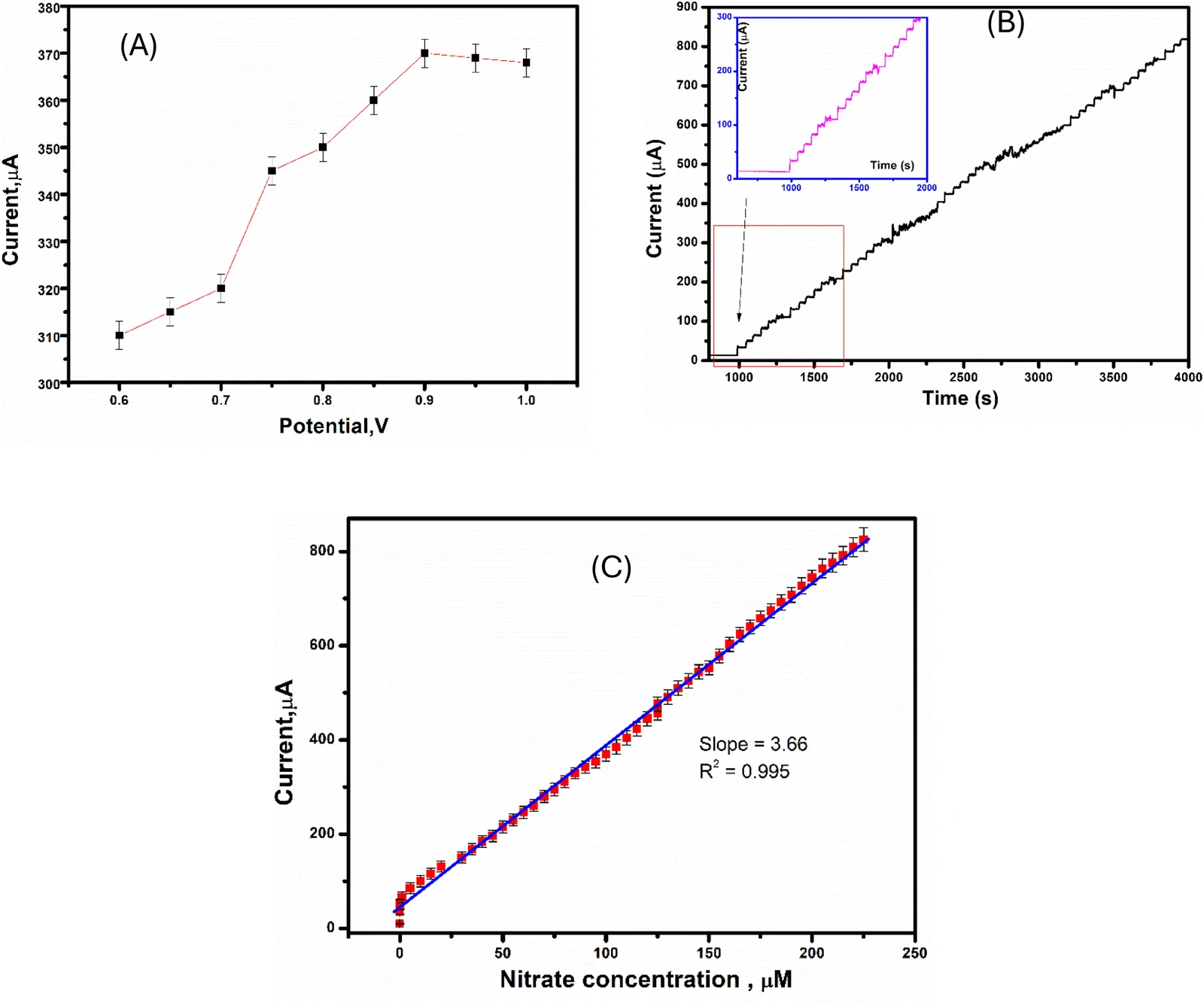
(A) Effect of different applied DC potentials (0.60–1.00 V) on the chronoamperometric oxidation response of nitrite ions at the TiO_2_–WO_3_@MWCNTs-modified electrode. (B) Representative chronoamperometric responses obtained upon successive additions of nitrite ions under the optimized experimental conditions. (C) Calibration curve showing the linearity between current response and nitrite concentration.

Chronoamperometric measurements were performed using the TiO_2_–WO_3_@MWCNTs/CPE under the optimized conditions for quantitative nitrite determination. Measurements were conducted in 0.1 M acetate buffer (pH 4.0) under continuous stirring, with successive nitrite additions at 50 s intervals. The applied potential was optimized between +0.60 and +0.90 V based on the preliminary chronoamperometric response (Fig. 8A). Each nitrite addition produced a rapid and well-defined current response, confirming the favorable electrocatalytic activity of the modified electrode. Among the investigated potentials, +0.90 V produced the highest amperometric response and the best linear correlation between current and nitrite concentration and was therefore selected for subsequent measurements. The chronoamperometric response at +0.90 V and the corresponding calibration curve are presented in Fig. 8B and C, respectively. The sensor exhibited a rapid response time of approximately 1.2 s and a wide linear range of 0.01–210 µM. The resulting LOD and LOQ were 0.003 and 0.009 µM, respectively, demonstrating excellent sensitivity toward low-level nitrite detection. As summarized in Table 2, comparison with previously reported nanomaterial-based electrochemical nitrite sensors demonstrates the competitive analytical performance of the TiO_2_–WO_3_@MWCNTs/CPE, particularly in terms of its low detection limit, broad linear range, rapid response, and high sensitivity. This enhanced performance can be attributed to the synergistic combination of the electroactive TiO_2_ and WO_3_ components with the highly conductive MWCNTs network, which promotes efficient electron transfer, increases the accessible electroactive surface area, and provides abundant catalytic sites for nitrite oxidation. These characteristics highlight the potential of the developed electrode for sensitive and rapid nitrite determination in food-safety applications.

**Table 2:** Comparative analytical performance of the TiO_2_–WO_3_@MWCNTs-modified carbon paste electrode (CPE) and previously reported nanomaterial-based electrochemical sensors for nitrite determination^a^

Nanomaterials modified electrode	Method	Linear range (mM)	LOD (mM)	Ref.
NiFe-LDH/Mo_2_C	—	0.005–260	1.7	51
Au@Ag/carboxylated graphene	CV	2.5–1250	150	52
Ni@Pt NPS/Gr	CA	10–15000	490	53
Au NPs/graphene-chitosan	CA	0.9–18.9	30	54
Chitosan/g-C_3_N_4_	DPV	40–2000	21	
	CA	20–4230	10	55
Iron sulfide nanorods-functionalized carbon cloth	CA	0.625–235.5	0.07	56
Molybdenum disulfide	CA	1–386	0.028	57
Tin sulfide	CA	0.1–2830	0.0136	58
Carbon spheres decorated with iron oxide/iron carbide/iron nanoparticles	CA	1–2540	0.06	59
Near spherical zin oxide	CA	0.6–5500	0.39	60
	LSV	1.9–5900	0.89	
Iron–nickel layered double hydroxide nanosheet arrays supported on carbon cloth	CV	2–1000	0.4	61
Silica/carbon/alumina/silica pyridinium chloride group	CA	0.2–280	0.01	62
Gold nanowires/carbon nano fibers/graphene	CA	1.98–3771	1.24	63
Ammonia oxidizing bacteria (AOB)	CA	7–70	2.39	64
		70–700		
a-Manganese oxide/molybdenum disulfide	CA	100–800	16	65
Flexible laser-induced graphene electrodes functionalized by CNT decorated by Au nano-particles	SWV	10–140	0.9	66
Bismuth selenide/carboxylic MWCNT	CA	0.625–77.5	0.002	67
		0.625–3387.5		
Molybdenum oxide/cobalt oxide/carbon cloth	CA	0.3125–4514	0.075	68
Hierarchical porous carbon, silver sulfide nanoparticles and fullerene	DPV	4–148	0.09	69
Metal–organic gel-carbon nanotube	DPV	0.3–100	0.086	70
Zinc oxide/Nafion	CA	0.36–1	0.21	71
	LSV	0.8–4.8	0.62	
Gold nanoparticles/cobalt hydroxide/carbon cloth	CA	0.625–77.5	0.1	72
Carbon selenide nano films on carbon cloth	LSV	0.5–1800	0.04	73
Silver/copper/MWCNT	CA	1–1000	0.2	74
ZrO2@MWCNTs	CA	5–100 µM	0.94 µM	75
TiO_2_–WO_3_@MWCNTs	CV	0.01–100 mM	0.003 mM	This work
	CA	0.01–210 µM	0.003 µM	

a Abbreviations: CA, chronoamperometry; DPV, differential pulse voltammetry; SWV, square-wave voltammetry; MWCNTs, multi-walled carbon nanotubes.

### 3.4. The mechanism of nitrite detection

The enhanced electrochemical response toward nitrite is attributed to the synergistic contribution of the TiO_2_–WO_3_ heterointerface and the highly conductive MWCNTs network. The TiO_2_ and WO_3_ components provide abundant surface-active sites that facilitate nitrite adsorption and interfacial oxidation, while the interconnected MWCNTs network promotes efficient electron transport and increases the electrochemically accessible surface area. The intimate contact between TiO_2_ and WO_3_ may further facilitate interfacial charge redistribution and charge-transfer processes. Together, these features provide a favorable microenvironment for nitrite oxidation and contribute to the enhanced analytical response of the TiO_2_–WO_3_@MWCNTs/CPE.

Under the applied anodic potential, nitrite undergoes direct electrochemical oxidation at the modified electrode surface according to the following overall reaction:NO_2_^−^ + H_2_O → NO_3_^−^ + 2H^+^ + 2e^−^

**Scheme 1:**
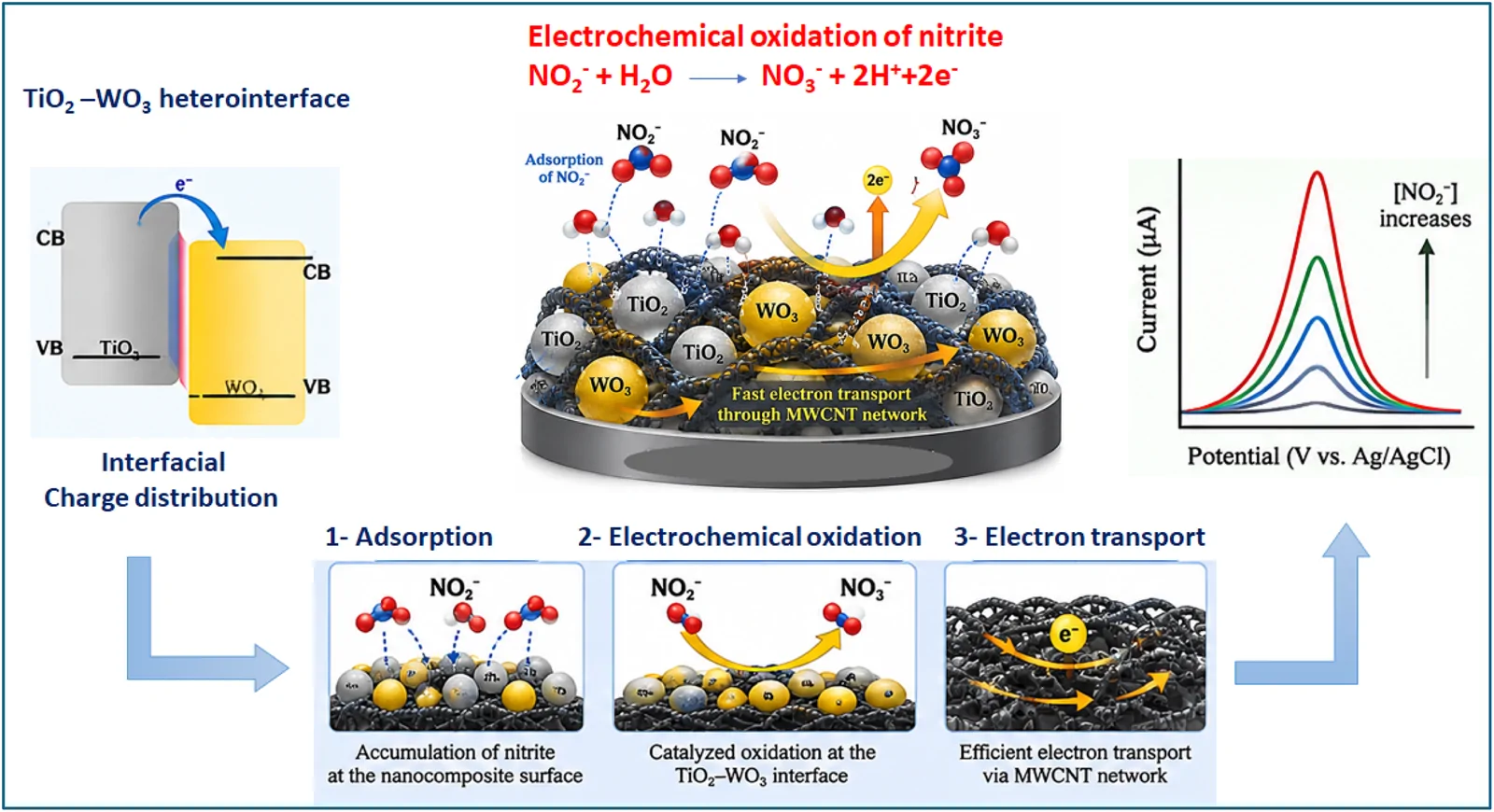
Schematic illustration of the proposed electrochemical sensing mechanism for nitrite oxidation at the TiO_2_–WO_3_@MWCNTs-modified CPE.

The generated electrons are efficiently transported through the highly conductive MWCNTs network to the underlying carbon paste electrode, thereby facilitating the faradaic oxidation process. The TiO_2_–WO_3_ heterostructure contributes additional electroactive sites and favorable interfacial properties, whereas the MWCNTs framework provides rapid electron-transfer pathways. The combined effects of enhanced nitrite adsorption, increased electroactive surface area, and improved interfacial electron transport account for the high oxidation current and improved analytical sensitivity observed for the TiO_2_–WO_3_@MWCNTs-modified electrode. The proposed sensing mechanism (Scheme 1) is based on the synergistic integration of surface-active TiO_2_/WO_3_ sites with the conductive MWCNT network, which facilitates nitrite accumulation, interfacial oxidation, and efficient electron transport, leading to enhanced electrochemical detection performance.

### 3.5. Selectivity testing of the nitrite sensor

**Fig. 9:**
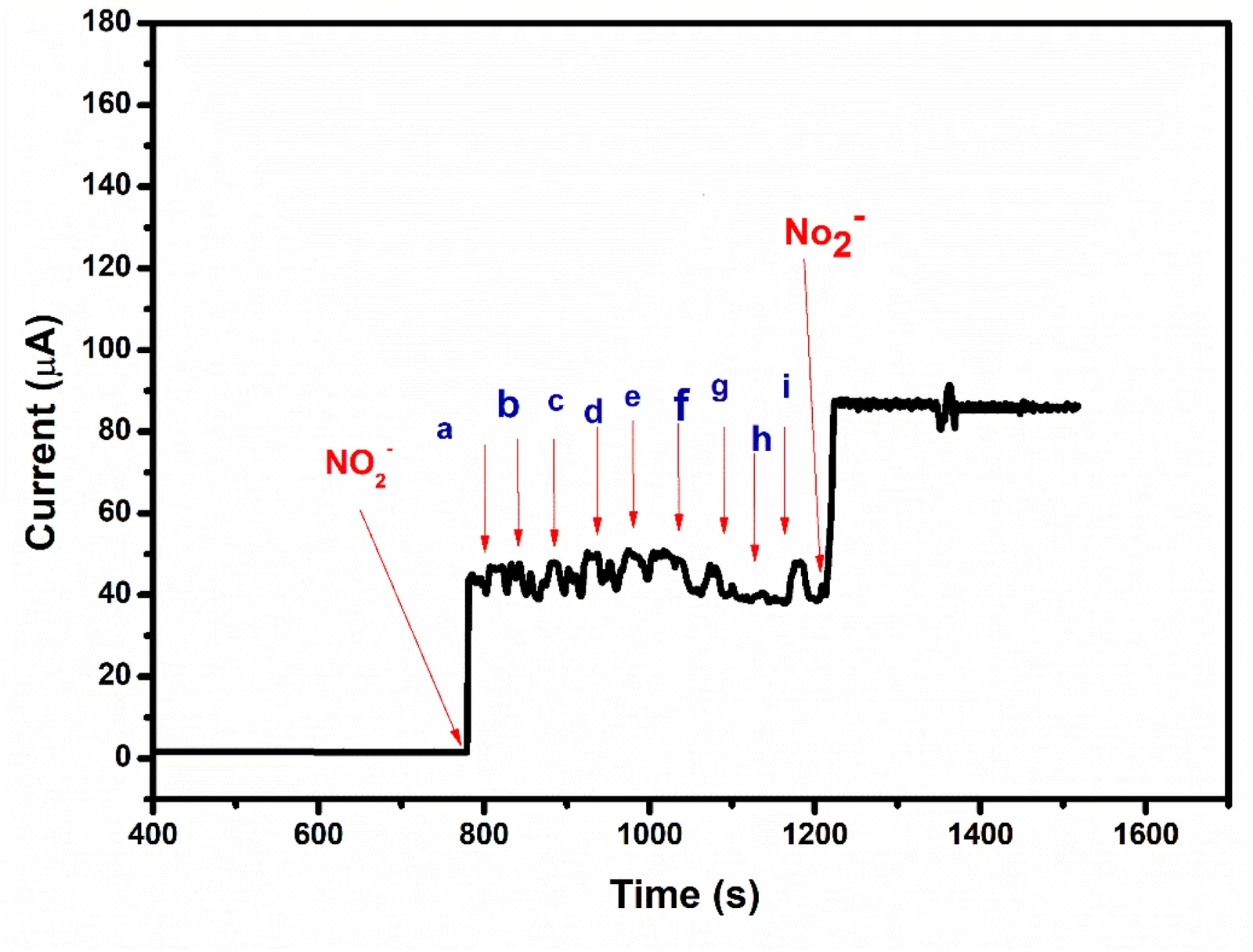
Chronoamperometric responses of the TiO_2_–WO_3_@MWCNTs-modified electrode toward the nitrite ions (0.5 µM) in the existence of various potential interfering species (0.1 M), including (a) ascorbic acid, (b) ammonium chloride, (c) lead nitrate, (d) d-glucose, (e) copper sulfate pentahydrate, (f) sodium chloride, (g) potassium chloride, (h) urea, and (i) copper chloride dihydrate. Measurements were performed following successive additions of nitrite ions (0.05 µM) into acetate buffer.

Since the developed electrochemical sensor is intended for practical analysis of real samples, it is essential to evaluate its selectivity in the existence of interfering substances commonly encountered in complex sample matrices. Therefore, performance of the TiO_2_–WO_3_@MWCNTs-modified electrode was systematically investigated against several common inorganic ions and food additives. For this purpose, 0.5 mM solutions of potential interfering species, including ascorbic acid, ammonium chloride, lead nitrate, d-glucose, copper sulfate pentahydrate, sodium chloride, potassium chloride, urea, and copper chloride dihydrate, were individually prepared and examined using chronoamperometric measurements under the optimum conditions. As illustrated in Fig. 9, the tested interfering compounds produced negligible electrochemical responses at the modified electrode surface, indicating minimal interference with the nitrite sensing process. In contrast, successive additions of nitrite ions generated rapid and pronounced increases in oxidation current, even in the presence of mixtures containing the foreign species. These observations demonstrate that the fabricated sensor possesses excellent selectivity toward nitrite ions and is highly resistant to interference from coexisting substances commonly found in food samples. The remarkable selectivity of the TiO_2_–WO_3_@MWCNTs/CPE owing to the synergistic electrocatalytic properties of the nanocomposite structure, which promotes preferential adsorption and oxidation of nitrite ions while suppressing nonspecific electrochemical responses from other species. The obtained results confirm that the developed TiO_2_–WO_3_@MWCNTs electrochemical sensor exhibits high selectivity, ultra-sensitivity, and fast response characteristics, highlighting its strong potential for reliable nitrite determination in complex real-sample matrices.

### 3.6. The sensor reproducibility and life time

The reproducibility of the fabricated TiO_2_–WO_3_@MWCNTs-modified sensor was evaluated through chronoamperometric measurements performed at a fixed nitrite concentration of 50 µM using several independently prepared sensor electrodes. The obtained oxidation current responses exhibited excellent consistency among the different electrodes, with a relative standard deviation (RSD) of approximately 3.65%, as presented in Fig. S1a. These findings confirm the high stability of electrode surface modification as long as high reproducibility and reliability of the electrode fabrication procedure.

Furthermore, the long-term stability and operational durability of the developed sensor were systematically investigated over a storage period of four weeks following fabrication. The sensor retained approximately 94% of its initial electrochemical response after the testing period, corresponding to only a 6.0% decrease in signal intensity, as shown in Fig. S1b.

The excellent reproducibility and stability of the TiO_2_–WO_3_@MWCNTs/CPE may be due to the robust integration of the metal oxide nanostructures within the conductive MWCNT network, which provides enhanced structural integrity, stable electron–transfer pathways, and strong adhesion of the active sensing layer onto the electrode surface.

### 3.7. Nitrite detection in real samples

The real application of the TiO_2_–WO_3_@MWCNT/CPE for nitrite determination was evaluated in processed meat samples using chronoamperometric measurements (*n* = 3), and nitrite concentrations were calculated from the incremental current response upon spiking with known standards. The sensor exhibited excellent analytical performance, with recovery range from 98.32% up to 101.48% and low relative standard deviations (0.9–2.9%), confirming high accuracy, precision, and matrix tolerance (see Table 3). These results demonstrate the reliability of the nanocomposite-based platform for direct, rapid nitrite determination in food matrices, highlighting its simplicity, and suitability for portable sensing applications.

**Table 3:** Determination of nitrite in real food and water samples using the newly developed TiO_2_–WO_3_@MWCNT-based electrochemical sensor, evaluated *via* the standard addition method, showing recovery rates and relative standard deviations (RSD%) under optimized conditions

Sample	NO_2_^−^			Recovery (%) *C*/(*A* + *B*) × 100	RSD (%) *n* = 3
	Detected ±SD (µM) (*A*)	Spiking (µM) (*B*)	Detected of total (detected + spiking) (µM) (*C*)		
Chicken salami	4.6 ± 0.5	50	54.0	98.90	2.9
	4.6 ± 0.4	100	104.3	99.71	2.1
	4.6 ± 0.5	150	154.1	99.67	1.3
Meat salami	4.04 ± 0.7	50	54.84	101.48	0.9
	4.0 ± 0.6	100	104.9	100.86	1.9
	4.0 ± 0.8	150	154.9	100.58	1.1
Sausage	4.9 ± 0.3	50	54.2	98.72	2.1
	4.9 ± 0.4	100	103.2	98.37	2.8
	4.9 ± 0.6	150	153.6	99.16	1.6
Burger	4.8 ± 0.7	50	54.9	100.18	0.7
	4.8 ± 0.3	100	104.2	99.42	1.2
	4.8 ± 0.5	150	154.3	99.67	2.4

## 4. Conclusion

A novel TiO_2_–WO_3_@MWCNTs-modified carbon paste electrode was successfully developed for ultrasensitive and high selectivity electrochemical platform for nitrite determination in food samples. The synergistic integration of TiO_2_–WO_3_ nanostructures with multi-walled carbon nanotubes significantly enhanced the electrocatalytic activity, conductivity, and charge-transfer efficiency of the sensing interface, as supported by CV and EIS analyses. The fabricated sensor exhibited excellent stability, reproducibility, and high selectivity toward nitrite in the presence of potential interferents. Under optimized chronoamperometric conditions, the sensor demonstrated strong analytical performance with high accuracy in real sample analysis, yielding satisfactory recovery values. The TiO_2_–WO_3_@MWCNTs/CPE platform provides a robust, cost-effective, and reliable approach for rapid nitrite determination. Future developments may focus on integrating the sensor with portable wireless potentiostat and smartphone-based readout systems to enable real-time food safety monitoring.

## Conflicts of interest

There are no conflicts to declare.

## Supplementary Material

RA-OLF-D6RA06391H-s001

## Data Availability

The authors confirm that the data supporting the findings of this study are available within the article. Supplementary information (SI) is available. See DOI: https://doi.org/10.1039/d6ra06391h.
