# Supplementary material for: Interfacial engineering of TiO2–WO3@MWCNTs nanocomposites for ultrasensitive and highly selective electrochemical nitrite sensing in food samples
Source: RSC Adv. 2026 Sep 3;16(43):52776–93. doi: 10.1039/d6ra06391h (PMC13540803; doi:10.1039/d6ra06391h)
Supplement: Supplementary file 1 [file RA-016-D6RA06391H-s001.pdf]

Supplementary Information (SI) for RSC Advances.
This journal is © The Royal Society of Chemistry 2026

Supplementary Information

Interfacial Engineering of TiO_2-WO_3@MWCNTs Nanocomposites for Ultrasensitive and Highly Selective Electrochemical Nitrite Sensing In Food samples

Asmaa H. Ragab[1], Hend S. Mager [2*], Rasha M. El-nashar[3], Rabeay Y. A. Hassan[1]*

[1]Biosensors Research Lab, Zewail City of Science and Technology, 6th October City, Giza 12578, Egypt.

[2] Applied Organic Chemistry Department, National Research Centre (NRC), Dokki, Giza 12622, Egypt.

[3] Chemistry Department, Faculty of Science, Cairo University, Giza 12613, Egypt.

***CORRESPONDING AUTHORS**

Hend S. Mager
Applied Organic Chemistry Department,
National Research Centre (NRC),
Dokki, Giza 12622, Egypt
Email: hendamer2000@yahoo.com

Rabeay Y. A. Hassan, PhD
Biosensors Research Lab,
Zewail City of Science and Technology,
6th October City, Giza 12578, Egypt
Email: ryounes@zewailcity.edu.eg
Phone: +201129216152

Table S1. Peak-to-peak separation (ΔEp) of the ferri/ferrocyanide redox couple at the TiO_2-WO_3@MWCNT -modified CPE as a function of scan rate.

**Scan rate ($V\ s^{-1}$)**	**ΔEp (V)**	**ΔEp (mV)**
0.05	0.19700	197.0
0.10	0.25764	257.6
0.20	0.32364	323.6
0.30	0.34660	346.6
0.40	0.34700	347.0
0.50	0.35289	352.9
0.60	0.35840	358.4
0.70	0.52760	527.6
0.80	0.56340	563.4
0.90	0.58726	587.3
1.00	0.62340	623.4

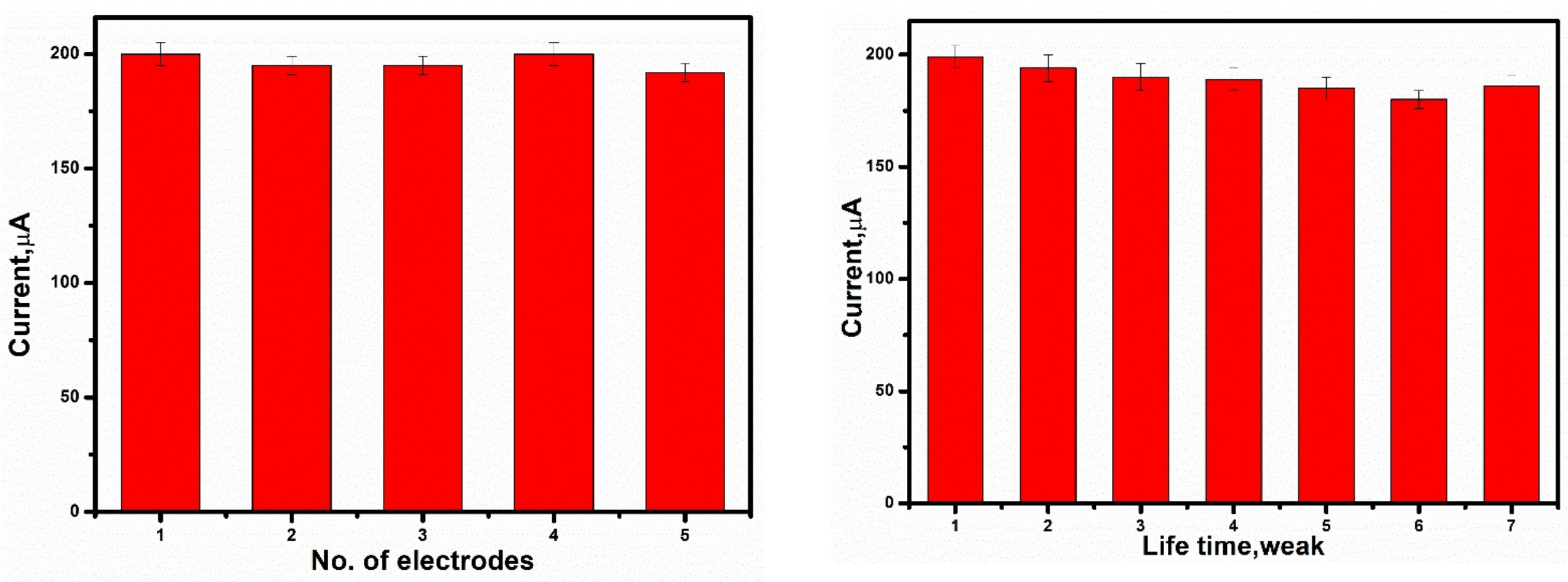

(A) **(B)**

Figure S1: (a) Reproducibility study of the TiO_2-WO_3@MWCNT-modified electrode evaluated over five independently fabricated electrodes using 50 µM nitrite under optimal conditions. (b) Long-term stability assessment of the modified electrode over several weeks, measured using 50 µM nitrite at optimized experimental conditions.
